# Antimicrobial and antifungal properties of the essential oil and methanol extracts of Eucalyptus largiflorens and Eucalyptus intertexta

**DOI:** 10.4103/0973-1296.66930

**Published:** 2010

**Authors:** Javad Safaei-Ghomi, Atefeh Abbasi Ahd

**Affiliations:** *Essential Oils Research Institute, University of Kashan, Kashan, I. R, Iran*

**Keywords:** Antimicrobial activity, *Eucalyptus largiflorens*, *Eucalyptus intertexta*, minimum inhibitory concentration, zone of inhibition

## Abstract

This study was conducted to evaluate the in vitro antimicrobial properties of essential oil, its major component, 1,8-cineole, and extracts of two *Eucalyptus* species, *Eucalyptus intertexta* and *Eucalyptus largiflorens.* Minimum inhibitory concentration (MIC) of the extracts was calculated by broth dilution method and the zone of inhibition was studied by agar disk diffusion method. Gentamicin (10 *µ*g/disk) and rifampin (5 *µ*g/disk) were used as reference controls for antibacterial studies and nystatin (100 *µ*g/disk) for antifungal studies. The results of MIC study revealed that the essential oil has a stronger activity and broader spectrum than those of methanol extracts. It is interesting to point out that the oils had even greater potential of antimicrobial activities than those of 1,8-cineole as their main component.

## Introduction

The genus *Eucalyptus* is known for its rich source of bioactive compounds.[1] It is a source for several unique secondary metabolites, which show a variety of biological activities, such as those of antioxidants, antibacterials, HIV inhibitors, attachment inhibitors, and others.[2–9] Although reports on the essential oil composition of different Eucalyptus species are relatively common,[10–15] investigations on their biological activities are still scarce. *Eucalyptus intertexta* and *Eucalyptus largiflorens* are two cultivated and adapted *Eucalyptus* species in warm regions of Iran, Kashan. To the best of our knowledge, the chemical composition of their essential oil is previously reported,[16–18] but there is no report on antimicrobial profiles of these two species. Thus, in this study, the *in vitro* antimicrobial activities of their essential oil, its main component and extracts, were evaluated against a set of 11 microorganisms. Their activity potentials were qualitatively and quantitatively assessed by the presence or absence of inhibition zones, zone diameters and minimum inhibitory concentration (MIC) values.

## MATERIALS AND METHODS

### Plant material

Leaves of *E. largiflorens* and *E. intertexta* were collected from cultivated sample in Kashan Botanical Garden (Isfahan Province, Iran) at an altitude of ca. 1000 m in December 2006.The voucher specimen of the plant has been placed in the herbarium of Kashan Research Botanical Garden, Research Institute of Forests and Rangelands, Kashan, Iran.

### Drugs and chemicals

1,8 Cineol was obtained from Sigma–Aldrich Chemie (Steinheim, Germany). Gentamicin, rifampin and nystatin were purchased from Himedia (Mumbai, India). Analytical grade methanol, dimethyl sulfoxide (DMSO), HPLC grade chloroform, anhydrous sodium sulfate, tween 40, and all culture media were obtained from Merck (Darmstadt, Germany). Ultrapure water was used for the experiments.

### Isolation of the essential oil

A portion (100 g) of dried and finally ground plant material was subjected, for 3.5 h, to water distillation using a Clevenger-type apparatus as recommended by European Pharmacopoeia.[19] The obtained essential oil was dried over anhydrous sodium sulfate and, after filtration, stored in amber vial at 4°C until analysis. The yield based on dry weight of the sample was calculated.

### Preparation of methanol extracts

A portion (20 g) of the powdered plant material was soxhlet-extracted with methanol for 8 h, at a temperature not exceeding the boiling point of the solvent. The extracts were concentrated using a rotary evaporator at 50°C to get crude extracts. Dried extracts were suspended in water and partitioned with chloroform to obtain polar (MW) and non-polar (MC) fractions. All the extracts were dried and kept in the dark at 4°C prior to use.

### Antimicrobial activity

Microorganisms

The essential oil was tested against 11 microorganisms including *Aspergillus niger* ATCC 16404, *Candida albicans* ATCC 10231, *Pseudomonas aeruginosa* ATCC 27853, *Bacillus subtilis* ATCC 6633, *Staphylococcus aureus* ATCC 29737, *Escherichia coli* ATCC 10536, *Klebsiella pneumoniae* ATCC 10031, *Staphylococcus epidermidis* ATCC 12228, *Shigella dysenteriae* PTCC 1188, *Proteus vulgaris* PTCC 1182 and *Salmonella paratyphi*-A serotype ATCC 5702. All were provided by Iranian Research Organization for Science and Technology (IROST). Bacterial strains were cultured overnight at 37°C in nutrient agar (NA) and fungi were cultured overnight at 30°C in sabouraud dextrose agar (SDA).

### Disk diffusion assay

The *in vitro* antimicrobial activity of samples was evaluated by the disk diffusion method (NCCLS).[20] The dried plant extracts were dissolved in DMSO to a final concentration of 30 mg/mL and filtered using 0.45 µm millipore filters for sterilization. Antimicrobial tests were carried out using the disk diffusion method[21] and employing 100 µL of suspension containing 10^8^ CFU/mL of bacteria, 10 ^6^ CFU/mL of yeast and 10 ^4^ spore/mL of fungi spread on the NA, SDA and potato dextrose (PD) agar mediums, respectively. The disks (6 mm in diameter) impregnated with 10 µL of the essential oil, a commercial sample of 1,8-cineole or the extract solutions (300 µg/disk) and DMSO (as negative control) were placed on the inoculated agar. The inoculated plates were incubated for 24 h at 37°C for bacterial strains and for 48 and 72 h at 30°C for yeast and mold isolates, respectively. Gentamicin (10 µg/disk) and rifampin (5 µg/disk) were used as positive controls for bacteria and nystatin (100 IU) for fungi. The diameters of inhibition zones were used as a measure of antimicrobial activity and each assay was repeated two times.

### Microwell dilution assay

MIC values were measured by microwell dilution assay method.[22] The inocula of the microbial strains were prepared from 12 h broth cultures and suspensions were adjusted to 0.5 McFarland standard turbidity. The samples were dissolved in 10% DMSO and diluted to the highest concentration (500 µg/mL) to be tested, and then serial twofold dilutions were made to a concentration ranging from 7.8 to 500 µg/mL in 10 mL sterile test tubes containing brain heart infusion (BHI) broth for bacterial strains and sabouraud dextrose (SD) broth for yeast. The 96-well plates were prepared by dispensing 95 µL of the culture media and 5 µL of the inoculum into each well. A 100-µL aliquot from the stock solutions of the plant extracts initially prepared at the concentration of 500 µg/mL was added into the first well. Then, 100 µL from their serial dilutions was transferred into six consecutive wells. The last well containing 195 µL of the culture media without the test materials and 5 µL of the inoculum on each strip was used as the negative control. The final volume in each well was 200 µL. Gentamicin and rifampin for bacteria and nystatin for yeast were used as standard drugs for positive control in conditions identical to tests materials. The plates were covered with sterile plate sealers. Contents of each well were mixed on plate shaker at 300 rpm for 20 s and then incubated at appropriate temperatures for 24 h. Microbial growth was determined by the presence of a white pellet on the well bottom and confirmed by plating 5 µL samples from clear wells on NA medium. The MIC value was defined as the lowest concentration of the plant extracts required for inhibiting the growth of microorganisms. All tests were performed in duplicate.

### Minimum inhibitory concentration agar dilution assay

MIC values of 1,8-cineole for the fungus isolate sensitive to it were evaluated based on the agar dilution method.[23] Appropriate amount of this compound was added aseptically to sterile, molted SDA medium containing tween 20 (0.5%, v/v) to produce a concentration range of 7.8-500 µg/mL. The resulting SDA agar solutions were immediately mixed and poured into petri plates. The plates were spot inoculated with 5 µL (10^4^ spore/mL) of fungus isolate. Nystatin was used as reference antifungal drug and the inoculated plates were incubated at 30°C for 72 h. At the end of the incubation period, the plates were evaluated for the presence or absence of growth. The MIC was defined as the lowest concentration of the compound needed to inhibit the growth of microorganisms. Each test was repeated at least twice.

## RESULTS AND DISCUSSION

Hydrodistillation of aerial parts of *E. largiflorens* and *E. intertexta* yields, respectively, 1.85 and 1.5% (w/w) of light yellowish oil.

According to the results given in Table 1, the essential oil of *E. intertexta* had great potential of antimicrobial activities against seven of the nine bacteria and a yeast species tested. Non-polar and polar fractions of the methanol extract were also found to be effective against one and four bacterial species, respectively, of the nine examined. However, essential oil and non-polar extract failed to show antifungal activity. The maximal inhibition zones and MIC values for bacterial strains which were sensitive to the essential oil, and non-polar and polar extracts of *E. intertexta,* were in the range of 10–12 mm and 7.8–15.6 µg /mL, 16–17 mm and 62.5 µg/mL, and 10–17 mm and 31.2–500 µg /mL, respectively.

**Table 1:** Antimicrobial activity of the essential oil, its major components (1,8-cineole) and methanol fractions of *E. intertexta* and *E. largiflorens*

Test microorganisms	*Essential oil*				Main component		Extract								Antibiotics					
	*E. intertexta*		*E. largiflorens*		1,8-cineol		*E. intertexta*				*E. largiflorens*				Rifampin		Gentamicin		Nystatin	
							CHCl3		H2O		CHCl3		H2O							
	DDa	MICb	DD	MIC	DD	MIC	DD	MIC	DD	MIC	DD	MIC	DD	MIC	DD	MIC	DD	MIC	DD	MIC
*Ps. aeruginosa*	—	—	—	—	—	—	—	—	10	31.2	—	—	10	31.2	—	—	23	500	NA	NA
*B. subtilis*	11	15.6	28	125	30	250	—	—	—	—	—	—	—	—	13	15.6	21	500	NA	NA
*E. coli*	10	15.6	—	—	20	500	—	—	—	—	10	125	—	—	11	500	20	500	NA	NA
*St. aureus*	11	7.8	12	250	18	500	—	—	—	—	—	—	12	7.8	10	250	21	500	NA	NA
*K. pneumoniae*	15	7.8	20	125	20	500	—	—	10	125	—	—	—	—	7	250	22	500	NA	NA
*St. epidermidis*	11	7.8	15	125	22	250	17	62.5	15	31.2	—	—	16	31.2	40	250	35	500	NA	NA
*Sh. dysenteriae*	14	7.8	—	—	15	250	—	—	—	—	—	—	12	7.8	8	250	18	500	NA	NA
*Pr. vulgaris*	12	7.8	11	7.8	11	62.5	—	—	—	—	—	—	10	125	10	125	23	500	NA	NA
*Sa. paratyphi-A serotype*	—	—	10	62.5	12	500	—	—	10	125	—	—	10	62.5	—	—	21	500	NA	NA
*C. albicans*	12	7.8	31	125	11	31.3	16	62.5	17	62.5	14	125	11	31.2	NA	NA	NA	NA	33	125
*A. niger*	—	—	22	125	—	—	—	—	12	500	12	500	—	—	NA	NA	NA	NA	27	31.3

— indicates no antimicrobial activity, ^a^DD (disk diffusion method), inhibition zones in diameter (mm) around the impregnated disks, ^b^MIC (minimal inhibition concentrations as µg/ml), NA (not applicable)

The results of the bioassay [Table 1] also revealed that the essential oil of *E. largiflorens* exhibited moderate to high antimicrobial activity against all the bacteria, yeast and mold tested, except three microorganisms, *Ps. aeruginosa, E. coli* and *Sh. dysenteriae.* The evaluation of methanol fraction indicated that polar fraction showed strong activity against 7 out of 11 microorganisms while non-polar fractions did not posses any inhibitory action against the strains evaluated except *E. coli.*

Based on these results, it is possible to conclude that the essential oil has a stronger activity and broader spectrum than those of methanol extracts.

The relatively high antimicrobial activities of essential oils are most likely due to the presence of compounds with antimicrobial properties. A number of compounds present in relatively high concentrations in the essential oils are known to have antimicrobial properties. Particularly worth noting is 1,8-cineole (eucalyptol), which accounted for approximately 70.2% (v/v) of the *E. intertexta*[17] and 37.5% (v/v) of the *E. largiflorens* essential oil,[16] and which has been found to possess relatively strong antimicrobial properties against many important pathogens and spoilage organisms.[24–26] These reports are further supported by our finding about 1,8-cineole which showed high inhibitory activities against *C. albicans* and *Pr. vulgaris* with MIC values of 31.3 and 62.5 µg/mL respectively.

However, a comparison showed that the oils has greater potential of antimicrobial activities than those of 1,8-cineole as their main component [Table 1]. Otherwise, other compounds such as limonene, α-pinene, *P*-cymene, and terpineol-4-ol, which have relatively strong antimicrobial activities,[27,29] may be responsible for this activity. Therefore, the synergistic effects of these active chemicals with other constituents of the essential oils should be taken into consideration for the antimicrobial activity.

Moreover, as indicated in a previous report about the other *Eucalyptus* species, gram-positive bacteria are more sensitive to the essential oils than gram-negative bacteria,[30] while our results did not show any selective antimicrobial activity on the basis of the cell wall differences of bacteria.
